# “You take care of people, people will take care of you”: Moral Economies and an Unpredictable Drug Market

**DOI:** 10.1371/journal.pone.0320423

**Published:** 2025-04-02

**Authors:** Erin E. Gould, Siddhi S. Ganesh, Anthony J. DiMario, Jimi Huh, Ricky N. Bluthenthal, Rachel Carmen Ceasar

**Affiliations:** 1 Keck Medicine, Department of Population and Public Health Sciences, University of Southern California, Los Angeles, California, United States of America; 2 USC Dornsife College of Letters, Arts and Sciences, Department of Sociology, University of Southern California, Los Angeles, California, United States of America; 3 Center for Tobacco Control Research and Education, Cardiovascular Research Institute, University of California, San Francisco, California, United States of America; Maulana Azad Medical College, INDIA

## Abstract

**Introduction:**

Fentanyl is the leading cause of opioid-related overdose deaths in the United States. Given the exogenous market shock of fentanyl and subsequent transition in the illicit opioid supply, our analysis sought to explore the social and relational experiences of people who use opioids (PWUO).

**Methods:**

We conducted qualitative interviews with 30 PWUO (n = 30) in Los Angeles, CA from July 2021 to April 2022. To be eligible for this study, participants had to report being 18 years of age or older and any self-reported opioid, cannabis, and injection drug use within the past 30 days. We used constructivist grounded theory to analyze the contexts that contribute to lived experiences surrounding opioid use behaviors within social networks.

**Results:**

Within an unpredictable drug market contaminated by fentanyl, participants reported: 1) avoiding opioid withdrawal symptoms by sharing financial and material resources within social networks, 2) securing and cultivating known, predictable social ties to prioritize safe/ safer supply of opioids, and 3) avoiding and mitigating risk of overdose fatality by using opioids within peer groups.

**Conclusions:**

Our findings emphasize that while peer support plays a critical role in safety within moral economies of PWUO, structural changes are needed to address the additional harms from an unregulated drug supply. Harm reduction interventions such as fentanyl test strip and naloxone distribution, as well as medication for opioid use disorders may improve safety. However, with a fentanyl-contaminated drug supply increasing risk for PWUO, safer opioid distribution of pharmaceutical-grade opioids and overdose prevention programs are needed to effectively address the burden of withdrawal, overdose, and fatality prevention within peer groups.

## Introduction

The third and fourth waves of the opioid crisis have been marked by widespread fentanyl disruption of the drug market [1]. Fentanyl is 30–40 times more potent than heroin, contributing to the highest overdose prevalence of all opioids [1], and is faster-acting and shorter-lasting [2], reportedly resulting in more severe withdrawal symptoms [3,4]. In this unpredictable fentanyl-dominated drug market, people who use opioids (PWUO) face several disruptions to their social network and barriers to maintaining safety and well-being including 1) increased pressure to intervene in overdose events for self and peers, 2) testing supply to prevent overdose, 3) sourcing contamination and disruption, and 4) supply shocks and heroin scarcity [1].

PWUO often report concern for accidental fentanyl ingestion [5] and personal and peer group fentanyl-related overdose [5,6]. PWUO may be reluctant to rely on emergency first responders as personnel may not be available to respond to overdose and/ or their involvement invites potential stigma and violence [7]. Reliance on social support provides a potentially beneficial mitigation of risk and concerns related to fentanyl.

Opioid overdose fatality prevention programming in the 1990s and 2000s focused on the advent of interpersonal rescue strategies such as naloxone administration [8,9]. Increasing PWUO’s role as peer naloxone administrators has facilitated better first response interventions and reduced feelings of powerlessness in communities where overdose risk is high [10]. Harm reduction strategies such as fentanyl test strip (FTS) or drug sampling strategies may be individual, but others such as naloxone administration are inherently peer intervention strategies as it is administered for other people rather than oneself.

Heroin was the most observed opioid in early overdose prevention programming in the 1990s and 2000s [8]. While the roles of PWUO in intervening to reverse opioid overdoses have been studied, fentanyl has reshaped these roles by increasing the risks of the opioid landscape and thus the burden on individual PWUOs to prevent and intervene in their peers’ overdose events. This paper uses the frame of moral economy [11,12] to examine how interactions among PWUO have been reshaped and intensified by the rapid takeover of fentanyl within illicit drug markets in Los Angeles. Originally formulated by historian E.P. Thompson [13] to analyze 18^th^ century English bread riots, moral economy denotes how feelings, sociality, moral norms, and other putatively “non-rational” forces can intervene in and regulate economic markets and attendant risks. While earlier formulations of moral economy emphasized the rejection of asocial market norms, namely through riot and resistance, subsequent developments have emphasized the role moral feeling and mutuality play in protecting individuals and communities from economic risks and vulnerability [13–17]. The frame has proved particularly useful for substance use researchers, who often point to the role of moral economy, and related concepts, in both lessening *and* heightening risks associated with illicit drug markets and substance use disorders. Within moral economies of unhoused PWUO, shared vulnerabilities emerging from shifting drug markets and mutual interdependencies of communities shape relationships and facilitate safer substance use [18]. Substance using behavior is shaped by social forces and as such, the role of social capital in peer groups is important to explore as it is a determining factor of safety among PWUO [11]. Overdose occurrences and risk are also shaped by mental health [19], which is influenced by social support systems. By focusing on PWUO’s perspectives on peer support, we expand the literature by highlighting how mutualistic strategies employed by PWUO to mitigate fentanyl-related health risks are reshaped amidst an increasingly unpredictable drug market.

## Methods

This research was conducted with 30 PWUO (n = 30) in Los Angeles, CA from July 29, 2021 to April 6, 2022. All study participants provided written informed consent for participation and publication. To be eligible for this study, participants had to report being 18 years of age or older and any self-reported opioid, cannabis, and injection drug use within the past 30 days (Table 1) [20]. We used these criteria to generate a convenience sample of participants. The University of Southern California Human Research Protection Program (IRB) reviewed and approved all study procedures (Study ID #: HS-18-00624). Additionally, a federal Certificate of Confidentiality protects this data due to the potentially identifiable nature of this dataset. We have changed participant names in the results to ensure anonymity.

**Table 1:** Participant sociodemographic characteristics.

Sociodemographic characteristics	Mean	Standard Deviation
**Age**	41.5 years	11.16 years
	**Count (n = 30)**	**Percentage (%)**
**Gender**		
Male	21	70
Female	8	26.67
Other	1	3.33
**Race/ Ethnicity**		
White	13	43.33
Latinx	9	30
Black	2	6.67
Native	2	6.67
Asian	1	3.33
Other	3	10
**Income level**		
Less than $1,000	12	40
$1,000 to $1,400	3	10
$1,401 to $2,100	6	20
$2,101 or more	9	30
**Housing Stability**		
Unstably housed	17	56.67
Stably housed	13	43.33

We conducted 60-minute interviews with participants (S1 Interview Guide). Data was analyzed in a series of iterative steps via constructivist grounded theory [21–23] that are described in more depth elsewhere [20]. Analysis consisted of reading transcripts generated from 60-minute interviews, documenting initial analytic findings, and developing a codebook which consisted of 26 thematic codes (S2 Codebook). The codes were conceptually grouped, and results were generated via blocks of coded text from all the interview transcripts using ATLAS.ti [24]. We analyzed and compared across the data to develop a conceptual theory to explain participants’ experiences. In this analysis, we focused on findings pertaining to social relationships and drug market unpredictability which yielded three results.

## Results

### Result 1: Preventing opioid withdrawal symptoms by sharing finances within social networks

Participants described withdrawal as a severely altering experience. Below, Ryan detailed how people manage withdrawal from different opioids:

*“China White... it’s never made me feel like I was dying, it’s never put me in a point where I would steal from my mom to go get heroin. It’s not like that. Withdrawal from fentanyl... people will steal their own stuff and pawn it, just to go get money to get well.” (Ryan)*

He saw withdrawal as an intensely vulnerable state intricately tied to finances. He described withdrawal from fentanyl as more painful than that of other opioids and connected this to behavioral changes that would not occur outside of this experience. Without strong social ties to leverage, having money was often the only way that participants were able to procure substances to manage withdrawal. When individuals didn’t have sufficient finances, they would steal or pawn to mitigate the painful symptoms of withdrawal. While Ryan was able to avoid this by using China White, he observed that this was not the case for those around him.

Participants described avoiding opioid withdrawal symptoms by sharing financial and material resources within social networks. Leo reported sharing finances with his peers so that he could obtain an adequate supply to stave off withdrawal. He noted the mutuality of this exchange and the importance of caring for those around him in this manner:

*“[N]ot having fentanyl is absolutely the worst feeling in the world...if I don’t have enough money... my friend will lend me some. He won’t let me be sick... You take care of people, people will take care of you.” (Leo)*

Given the potency of fentanyl and severity of its withdrawal, keeping an adequate supply was a major concern for participants. Due to changes in financial circumstances, money-making strategies, and market-driven opioid price fluctuations, PWUO in our study did not consistently have the financial means to maintain a supply of opioids for themselves. Many participants in our sample were economically marginalized, with the vast majority having an income level less than $2,101 a month. Relying on peer support, where individuals can pool resources together when one individual is lacking, played a critical role in alleviating the threat of withdrawal.

Participants noted the difficulty sourcing drugs during transitional periods. For instance, Lucas had one individual he bought drugs from both for personal use and to distribute himself for money, his “plug,” before moving cities. However, following his move to Los Angeles, he ran out of money and was in withdrawal. At that time, he reached out to his plug because he did not have enough money to obtain opioids in this new location:

*“And then, by the time I got to LA, I had a little bit of money left and like no drugs left. So I didn’t know what to do for money when I got here, so I ended up calling my [plug] up in [my previous city]. And we had a good relationship. I made him a lot of money. So I pretty much asked him if he could [lend] me some money. And he wired me like $1,000 and told me I didn’t have to pay him back, just good to go.” (Lucas)*

Lucas described this relationship with someone who was both his dealer and someone he sold for in the past as positive due to their lack of financial tension. Lucas also highlighted the fluidity between the roles of buyer and seller within the illicit opioid market, and how social connections influence this. The ability to rely on these drug-using peer networks represented a socially situated economic safety net that buffered the effects of withdrawal for PWUO. While moving locations represented a level of instability due to needing to find new drug sourcing connections alongside the financial burden of relocation, peer groups helped to mitigate the intensity of this.

Others described sharing drugs within their social circles to avoid withdrawal symptoms. While this was a successful strategy to avoid withdrawal symptoms, Ruby noted various feelings including guilt and anxiety that accompanied relying on friends:

*“I got friends that’ll give me some [opioids] if I’m sick. They’ll give me some if I absolutely need it, but I try not to get it from them. It’s not fair [to them] to have to keep me in my habits... that’s my job that’s my habit. I brought it here and so I always like to take care of [myself on] my own. So that’s why I don’t do the partying with my friends... so that I can have enough money to get me high for the month.” (Ruby)*

Ruby expressed guilt about managing substance use on her own and attempted to structure her routine and finances with that in mind. While avoiding withdrawal was a motivating factor for peer reliance, the moral debt associated with sharing drugs within peer groups complicated this. Participants reported feelings of discomfort as this put them in a dependent position within their peer group. Further, for many PWUO, reliance on peers necessitated both giving and receiving support, the former of which may not have beeen accessible.

Maria recognized withdrawal was a vulnerable state to be in which could be exploited to get what you want:

*“My brother he was all strung out and I used to always see him fix and I used to always see him nod and feel so good. And one day I seen him... he was dope sick. And I had some monies and he didn’t [LAUGHS] and he needed to get well. And I told him the only way I was going to give him any money is if he gave me some because I wanted to try it.” (Maria)*

Maria described an experience where her brother was in withdrawal and acknowledged that as an opportunity to ask him to initiate her opioid use, which he otherwise would not have been open to. However, because he was in a painful state of withdrawal, he was willing to acquiesce to Maria’s request if that would abate his symptoms. In this case, social ties could be leveraged for personal gain. This illustrates the intense vulnerability associated with withdrawal, severe pain driving engagement in behaviors that PWUO otherwise would condemn, and power dynamics that exist within moral economies.

### Result 2: Cultivating and securing known and predictable social ties to prioritize steady supply of opioids

Participants sought out, developed, and maintained social connections in order to get stable supplies of opioids. Emery attributed the continuity of her heroin supply during the market shift to fentanyl to her network:

*“I haven’t had to deal with the loss of availability. I have several different connections where I can get it. I try to keep all three alleys open, because you never know who’s not gonna be there the next day. If they get busted or if they die or whatever is gonna happen to them. So, I have to keep a good three options open. And what I mean by three is probably nine people I can get it from. But I mean white people, Black people, and Mexican because those are the ones that are getting it from different people, they’re getting it from different connections. Like the Latino connections, they will bring it over themselves and then give it to their people to sell and the gang members or whatever. And I don’t know where the Black people get it, but they stay and they keep it in their own and it’s usually not the best quality. And then the white people that I get it from are usually getting it from bikers and it’s usually cross-contaminated with fentanyl so I try not to use that. But that’s basically the way it goes. I’m getting it.*” *(Emery)*

For Emery, access to “trusted” suppliers hinged on availability distributed across different groups. She reported that each group employed different strategies for sourcing which resulted in varying levels of quality and contamination. She noted how despite the unpredictability in the market, her strategic maintenance of open connections with various people prevented disruptions in supply

She further described observations about the transition to fentanyl as experienced by other people in her social network. She relied on these observations to make decisions about avoiding fentanyl because if she used fentanyl-contaminated products she would not be able to “stay well” over the long term:

*“[I]t seems like the people that went to fentanyl, heroin doesn’t help them get well anymore and there is nothing that can help them feel well. It’s like a heroin addict can do a pill or smoke a joint and pass the time until they get some, but fentanyl users can’t do anything. It’s just pain and it gets worse and worse and worse until they get it.”* (*Emery)*

These social ties took on increasing importance in an unpredictable drug market. Because of her diverse peer network and ability to find a consistent product, she could avoid unwanted, potentially contaminated sources, as well as mitigate symptoms of withdrawal which she would face without adequate availability.

Participants who knowingly sourced fentanyl also cultivated stable connections and sources. Theo noted that due to having a steady dealer he was able to obtain enough fentanyl even when his finances would otherwise complicate this:

*“I’ve stayed steady with the same dealer the whole time I’ve been doing fentanyl. So for like four years, I’ve gotten it from the same connect through this whole time. [It is advantageous because] if I need a front, I can get a front. Like my bags are fatter. Plus, he’s my friend. There’s two of them. They both ended up being really cool people. So the main benefit is I made a couple of really good friends.” (Theo)*

Moral economies of PWUO are sources of both substances and sociality. While a steady supply of fentanyl is imperative to avoid withdrawal symptoms, Theo also highlighted the social aspect of using the same dealer, which provided a crucial support system. He described these individuals as friends who could offer companionship, moral support, and people to use fentanyl with as he saw it as a “social drug.” Additionally, these relationships with suppliers were paramount for PWUO when experiencing economic strain. It was easier to weather financial changes due to these social bonds driving suppliers to prioritize interpersonal rather than simply economic gain.

Ruby similarly described keeping multiple sources of opioids open with the understanding that her known dealers could be arrested at any time and that would disrupt her access to supply:

*“[Y]ou have a connection when you are home you want to get high. You always know where to go get it and if they go to jail you have a backup. You have someone else you can go to.” (Ruby)*

Criminalization disrupted the stable supply of opioids sourced from an unregulated drug supply by taking away known channels of sourcing when sellers were incarcerated. When individuals were incarcerated, PWUO had to seek out new (and possibly unknown) dealers and sources, inviting the possibility of encountering fentanyl adulterated and substituted heroin (FASH).

Another participant relied on primary and secondary sources to avoid a loss of opioid availability. Isaac noted that to find these sources, he turned to his peer groups for trusted recommendations:

*“[In my location] most of us probably know each other and we’re getting from the same few people that sell it. You have to find your person, and you have to stick with your person. Matter of fact, you keep a backup person too. And so, you have a person, and you have a backup person. But if you lose them... I’ll just go down the line of friends who use and see if they have somebody.” (Isaac)*

Isaac described approaching cultivating social ties around sourcing opioids systematically and methodically. This was often due to an underlying anxiety about what would occur should he lose this connection.

With disruption to a steady supply coming from both market forces (i.e., proliferation of fentanyl in the illicit opioid market) and the criminal justice system (i.e., arrest of sellers), PWUO reported needing to seek out and develop social connections to maintain sources of opioids. They described how despite the shifting drug landscape, they manufactured a sense of stability via their social ties. Participants in this sample were largely unstably housed and had incomes of less than $2,101 per month. They had to simultaneously navigate material and drug market precarity. Because of the established trust between PWUO and their social networks, including suppliers, they were able to weather economic strain and loss of availability in unpredictable markets.

### Result 3: Mitigating risk of overdose fatality by using opioids with peer groups

Ruby avoided fatal overdoses by using opioids in the presence of peers equipped for overdose reversal:

*“[I use opioids] with my friends... I don’t like to get high alone because I’m afraid if I do too much or something then I’m not going to be able to make it out if nobody’s there with me so... I got the Narcan at the house and everybody knows where it’s at, we know how to use it... because you never know with the Fentanyl.****”*** *(Ruby)*

She reported that she felt wariness regarding the prevalence of fentanyl in the market within the fourth wave of the opioid epidemic in the 2020s. Ruby avoided using opioids alone because with others around her who could administer Narcan if need be, she felt safer despite possible fentanyl contamination.

Ruby described having a robust friend group and strong boundaries within it:

*“I got a lot of friends, some of them smoke, some of them don’t. Most of my friends use drugs but they don’t smoke pot. I got a lot of friends and everything, thank God. You know, you don’t have someone smoking in your house, smoking in my house. If it bothers you, just step outside for a minute... because it’s my house and I’m going to do what I want in it.” (Ruby)*

Having a strong and abundant social network was a source of security for Ruby. Importantly, she had clear boundaries around how and in what circumstances she would allow friendships to shape her ability to use drugs in ways she wanted to. Because her friends respected these boundaries in addition to being physically present and using opioids with her, she felt confident that she could lean on her social network for support. This allowed Ruby to reduce her fears about continuing to use opioids within a fentanyl-flooded market.

Ryan described an experience of unknowingly using fentanyl with friends. Following his overdose, emergency service personnel were disinclined to revive him. Instead, his friends intervened to provide Narcan and chest compressions, which were able to revive him:

*“I didn’t know what fentanyl looked like, I didn’t know what to look for, and it was dark, I wasn’t paying attention, and I didn’t look. And I did enough fentanyl that the paramedics said I was dead on arrival. They didn’t want to work on me. They’re like, ‘Nah, the dude’s dead. If he’s been like this for six minutes, no he’s gone.’ And my friends were like, ‘We’re gonna give him one more Narcan, fuck you, guys.’ They’d been doing chest compressions on me, and the paramedic’s like, ‘Well here, let us take over.’ They did two chest compressions, and they put the air thing on my mouth and [SNAPS]. Came back.” (Ryan)*

PWUO are often hesitant to involve emergency service personnel. This is due to fears that it could invite stigma, violence and/ or subject individuals to the carceral system. In this landscape, peer group reliance often serves as the only viable intervention strategy to avoid overdose fatality. Ryan also highlighted the increased risk of overdose with accidental fentanyl use. Due to its higher potency, people who use heroin may experience overdose and/or fatality when using fentanyl unaware.

Ryan described being consistently exposed to substances via friends across his lifespan. In childhood, he initiated cannabis and opioid use after introduction to it from members of his social network. He found cannabis to be a familiar substance because everyone in his family used it growing up. He emphasized that from childhood to adulthood, socializing with his network involved cannabis and/ or opioid use. While his friend group was a source of exposure to substances, they were also a driving force of safety. Because he had a strong social network and friends present while using opioids, he was able to be revived after overdosing on fentanyl.

PWUO expressed variability in the ways in which they relied on peer support. Isaac described his reliance on both friends and additional programs to avoid fatality:

*“I’d say [I use] about 75% of the time on my own, 25% [with friends]... All my friends say it scares them that I’d be alone by myself most of the time when I’m doing it... at the needle exchange they had this thing that says, 'Don’t shoot up alone.'... And I was like, 'Oh, this is really cool.' You call people before you shoot up, and then they talk to you while you’re doing [it]. And they call to check on you afterwards. And if you don’t respond, then they send the paramedics to your location. And I was like, 'This is perfect,' ‘cause there’s been a couple times where I shot up alone, and I woke up six hours later, like, 'What the fuck happened?' I don’t know how close I could have been. But to be asleep that long in a comatose state is probably closer than I want to be.” (Isaac)*

While Isaac had friends who he was able to use opioids with, he often preferred using opioids alone. As such, he felt that having overdose prevention interventions that implemented the protective aspects of peer support such as check ins and overdose response but did not require him to change his preferred ways of using substances allowed him to remain safe while not changing his routines.

Isaac went on to explain his motivation to largely use opioids on his own was driven by mistrust of his social network:

*“Even people that act all like my friends have stolen from me. I don’t do none of that stuff. That’s rare you find somebody that don’t. A lot of people are going to say they don’t, but... in the streets, my name is good. I’ve had girlfriends that I’ve been with that I give them everything...[heroin] makes people fucking crazy, man! They don’t care about nothing else... For that fucking drug, they’ll steal from me... I’ll give it to you. Why don’t you just ask?... You can’t trust nobody, not one bit... I tried to, but every single time I’ve gotten burned. And so, I keep everybody at a distance.” (Isaac)*

Even when discussing heroin, an opioid with a lower cost and less painful withdrawal experience as compared to fentanyl, individuals in Isaac’s life stole from him to procure it. Because this happened to him repeatedly, he was hesitant to rely on anyone. Fentanyl often intensified these dynamics. When individuals had weak social ties, mistrust led to lack of reliance on moral economies. This presented a significant safety issue as one important method of avoiding overdose among PWUO was unavailable.

## Discussion

Our findings show that in an unpredictable drug landscape marked by a fentanyl takeover of the illicit opioid supply, people are actively building new and leveraging existing social networks which can counter this unpredictability. Social support systems involved direct action to keep each other safe, which included 1) sharing finances to head off withdrawal effects and mitigate financial changes which hindered opioid access, 2) sourcing from known and trusted people to avoid disruptions to and contamination of supply with fentanyl, and 3) preventing overdose by using with peers, monitoring, and administering overdose reversal agents.

The frame of moral economy is useful for understanding these collective risk mitigation practices. Moral economy situates interpersonal transactions within a moral framework as well as an economic one [13]. This framework explains how moral and collective feelings about the ways in which markets should operate, often contravening values such as self-interest and profit, structure how actual commodities markets do operate. For example, Liang and Richardson [11] analyze the practice of “fronting” that is, provision of drugs on credit or for collateral, as essentially a moral economic practice that leverages participants’ social capital for material resources that may be protective in the short term, but can also expose recipients to potential harm in the form of unregulated and informal debt, violence, and damaged reputation.

We observed that within moral economies of PWUO, systems of exchange of goods (i.e., money, FTS, naloxone) and services (overdose intervention support) were in place to alleviate the strain of an unstable drug market. Sharing finances, substances, and injecting materials are common practices among PWUO [18]. Our data supports prior research indicating that social capital and proximity within peer groups are influential in determining support between dealers and buyers [11]. However, our findings indicate that these strategies take on increased significance in an unpredictable drug market. Existing data demonstrates that there are faster and more severe withdrawals due to fentanyl [3,4,25]. Participants in our sample feared FASH as well as intentional fentanyl use due to the risk these substances posed for dependency, withdrawal, and overdose. Because of this, we observed an increased need to mitigate effects by relying on peer networks and pooling resources. Withdrawal is a debilitating, sometimes fatal, experience comprising a multitude of physical and psychological symptoms not limited to nausea and vomiting, muscle aches and pains, anxiety, and insomnia [3,4,25]. PWUO report in prior data that its painful experience and influence on behavior to avoid it is often minimized or misdirected at individual characteristics [25]. Within a fentanyl-contaminated drug market, PWUO may have more difficulty avoiding withdrawal and overdose due to the need to use more frequent and higher quantities of fentanyl as compared to heroin [25]. Reliance on moral economies of PWUO is one safety strategy to minimize these risks. Despite market shifts, individuals with sufficient social support could lessen the impacts of precarity.

In result 1, participants in our study reported that the ability to manage withdrawal hinges on having the financial means to procure opioids. When this was not feasible, they relied on peers to support them financially so that they could use enough opioids to mitigate withdrawal symptoms. Prior research [25] supports our findings that PWUO rely on peer networks to share opioids or find connections to drug sellers if prior ones were unavailable to avoid withdrawal. Frank and colleagues [25] found that this reliance on peer groups reduced risk behaviors such as illegal money-making strategies or using drugs from unknown sources, which may be contaminated with fentanyl.

Among people who use heroin, participants in result 2 reported on the significance of sourcing uncontaminated opioids from known social ties in a fentanyl-flooded market. Accidental fentanyl exposure is common among PWUO. In one study, despite many opioid-using participants reporting that they did not use fentanyl, nearly 90% tested positive for fentanyl [26]. These results echo findings in the existing literature [5]. Lorvick and colleagues found that participants feared the risk of accidental overdose and wanted to change suppliers upon learning that they had used FASH [5].

Using within peer groups (i.e., “never fix alone”) as an established safety strategy to avoid overdose [18]. Our findings from result 3 demonstrate that this safety strategy has increased urgency with the higher likelihood of overdose and fatality associated with fentanyl. By keeping overdose reversal agents on hand and ensuring that social groups have the skills to administer them, PWUO avoid fatality in situations where they encounter FASH.

In the current landscape, moral economies of PWUO are taking on the burden of risk reduction in lieu of other individuals or systems intervening [27,28]. They are acting as first responders when the alternative involves law enforcement, prohibition enforcement, and the potential for state sponsored violence [28,29]. In many settings, PWUO are unaware of good Samaritan laws which aim to protect them from retribution should they alert emergency services of overdose events [30]. PWUO report fear that involving law enforcement in overdose events, even in a landscape with good Samaritan laws, will result in arrest due to prior legal involvement (i.e., outstanding warrants, drugs and/ or drug paraphernalia possession) and displacement if they are unhoused [31–34]. Fears from PWUO stem from prior mistreatment by police due to unhoused and/ or substance use status [32]. In one Los Angeles study, law enforcement individuals reported that they responded to approximately two thirds of overdose events and made an arrest in 14% of them [35]. This data on criminal justice involvement demonstrates the critical need for interventions which PWUO can rely on to mitigate peer intervention burden without the risk of being criminalized.

Harm reduction-based peer support efforts are examples of prevention situated within the context of social support [36]. Participants in our study reported experiencing the need to prevent, intervene, and act as first responders in opioid overdose events. While peer support plays a critical role in safety and is often reported among PWUO, it results in moral debt that anticipates further reciprocity [11,36]. PWUO who receive support from their social networks are more likely to provide that support in turn [36]. However, these actions hinge on having the means to support those around you, which participants in our study at times did not have access to. Further, burnout among peer workers (PWUO with additional overdose prevention training) is a prevalent issue [37]. Within overdose prevention sites, peer workers are a critical component of the intervention [36,37]. They promote service engagement among PWUO due to improved interpersonal communication and increased feelings of safety [37]. However, burnout among peer workers results due to inadequate compensation, lacking support systems, grief and trauma related to witnessing overdose events and fatalities amidst and overdose epidemic, and personal experiences of financial and housing precarity [37]. Successful relationships between PWUO and peer recovery specialists also hinge on alignment between life stages and experiences [38]. Thus, increased provision of resources and funding support are warranted to address interpersonal and institutional barriers to providing adequate recovery and safety support to PWUO [37,38].

Direct services such as FTS and overdose reversal agent distribution alleviate the degree of harm participants experience by enabling PWUO to intervene when needed [39]. Having accessible supplies allows PWUO to prevent and/ or treat overdose from fentanyl-contaminated opioids within their social networks, which participants in this study described as efforts they are already engaged in. Employing peer-based naloxone distribution may be a way to strengthen moral economies, but myriad barriers to intervention exist. Lack of resource access [8] due to provider stigma, lack of training, unawareness of prescribing legislation [39], and interstate disparities [40] prevent PWUO from obtaining FTS and opioid reversal agents. Additionally, knowledge of use [8] is minimized by changing naloxone doses, shelf life, [41] and training requirements to effectively use it. Finally, time-based pressures to mitigate or avoid opioid withdrawal [18] are in opposition to attempts for safe consumption [42]. These factors complicate the capacity for peer intervention by needing to obtain materials and education. Ongoing efforts are needed to increase access to intervention strategies such as FTS and overdose reversal agents and destigmatize their procurement to mitigate overdose risk among PWUO.

Institutional-level interventions include medication for opioid use disorder (MOUD) in the age of fentanyl [39,43]. While participants in this study did not directly report MOUD use, these further reduce the burden on PWUO by lowering the chances of uncontrolled withdrawal as well as overdose and thus the need for direct intervention within social networks. However, medication-based treatments have limitations such as not being desired by or appropriate for all PWUO (i.e., due to lack of healthcare accessibility or use pattern incompatibility) resulting in underutilization [39]. Due to increased suitability, potency, and availability individuals may instead turn to illicit options [39] which pose risk to peer groups due to likelihood of FASH use and ensuing withdrawal and overdose risks.

Because of these limitations, policy-level changes are warranted and findings from this study have important public health and policy implications. Data from result 1 (Preventing opioid withdrawal symptoms by sharing finances within social networks) shows how withdrawal has important financial implications, especially given the higher cost and shorter half-life of fentanyl [1,2], and how participants relied on moral economies (i.e., networks of friends and peers) to share money and reduce withdrawal occurrence. Data from result 2 (Cultivating and securing known and predictable social ties to prioritize steady supply of opioids) shows how participants build and access social networks within moral economies to source a steady and potentially more reliable dose and composition of opioids amidst a fentanyl-flooded market. Data in result 3 (Mitigating risk of overdose fatality by using opioids with peer groups) shows how participants relied on moral economies for safety assurance when potentially using FASH. Despite the variance in strong or weak social ties within moral economies, they acknowledged that the unregulated drug supply posed a larger threat and there were still high rates of overdose and associated risks.

Policy support for less restrictive prescribing laws, overdose prevention programs (OPPs), and safe/ safer supply are imperative to adequately mitigate overdose occurrence and fatality [39] as well as the social strain from the need for peer intervention (as indicated by result 3). Safe/safer supply and OPPs are two policy interventions to mitigate major risks associated with the unregulated drug supply [39]. Safe/safer supply refers to prescribing pharmaceuticals as an alternative to unreliable substances within the illicit market [39]. This is done to reduce the risk of overdose and other complications from using contaminated, unregulated, and unwanted substances.

OPPs are highly effective at preventing overdose. Participants needed strong social networks to reduce overdose risk and maintain social constancy. OPPs can support this by reducing the moral burden placed upon those intervening in the current opioid crisis [37]. Increasing access to safe consumption sites may reduce consequences of substance use such as overdose and infection while also connecting PWUO to healthcare, social services, and substance use treatment [44]. Implementing radical hospitality (i.e., developing positive relationships with guests via compassionate interactions) within safe consumption sites has been found in prior research to promote positive sociality and protect safety via reducing overdose fatality within groups of PWUO [44]. However, with a fentanyl-contaminated drug supply increasing risk for PWUO, other harm reduction measures are often insufficient to effectively address the burden associated with withdrawal, overdose, and fatality vigilance within social networks. As such, safer opioid distribution of pharmaceutical-grade products is needed [39]. This is further called for to alleviate the strain on finances and difficulty sourcing that our data presents in results 1 and 2 respectively.

We found that participants shared finances to reduce risk of withdrawal and overdose [25]. All of these programs will make accessing drug supplies, withdrawal management, and overdose prevention medication more affordable and accessible via policy interventions.

Low-barrier distribution sites are already in practice, such as at safe consumption sites [45] and opioid vending machines [42] in Vancouver, Canada. That these efforts are already successfully in place indicates the importance not of implementation resources but of community, medical, and governmental support [39,45,46]. We observe a successful history of safe/ safer supply in other countries as well. In Liverpool, England, individuals with decades long heroin prescriptions were medically and socially stable [47,48]. When the clinic was defunded, patients returned to accessing heroin via the illicit market and experienced houselessness, overdose, and other negative ramifications. When Switzerland implemented heroin-assisted treatment (HAT), drug-related deaths decreased significantly from 5.3 to 1.6 per 100,000 residents [49]. Germany also saw improved physical and mental well-being among those engaged in HAT [50].

The combined availability of safe/safer supply with OPPs could reduce risks associated with not knowing the dose and combination of the opioid and the ability to intervene meaningfully in case of overdose. Implementation of safe/ safer supply and OPPs meets the safety needs of PWUO while also serving to decrease drug market-related violence, increase well-being [39], and alleviate some of the demands faced in social networks [51]. While there is still work to be done to improve safe/ safer supply and other OPPs, these efforts are important harm reduction practices which improve social interconnectedness, community, and optimism [51] to improve health outcomes alongside social relationships.

### Limitations

This study has limitations. This was a convenience sample with data collected at a methadone clinic and a syringe exchange site so it might overrepresent those engaged in treatment and harm reduction practices such as reliance on peer groups alongside FTS or naloxone use. This study also did not use anamnestic data regarding reinitiation or cessation of substance use. Inclusion included self-reported opioid, cannabis, and injection drug use within the past 30 days. As themes were inductively generated from the data and reflect the perspectives of participants, which, for the purposes of this analysis focused on moral economies and social networks, they did not pertain to reinitiation. Instead, this analysis focused on interpersonal relationships as they relate to sharing resources, sourcing, and safety strategies. Next, in result 2 (Peer Networks and Risk Mitigation) participants described cultivating social ties to prioritize steady opioid supplies. For example, Emery described how socioeconomic and racial factors influenced access to “trusted” suppliers. While we posit that there were likely variations in access to preferential opioids due to socioeconomic status, racialization, and neighborhood factors we did not specifically collect data to explore these distributions. Future research should examine how relationships with suppliers evolve under economic strain and how trust is established or broken in unpredictable markets. Additionally, future research should examine gender-based differences as they relate to peer reliance and social networks. Despite these limitations, this research provides critical nuance on the changing and unpredictable drug environment due to the presence of fentanyl from the perspectives of PWUO who are greatly impacted by it.

## Conclusion

Our findings emphasize that while peer support plays a critical role in safety within moral economies of PWUO, structural changes are needed to address the additional harms from an unregulated drug supply. Harm reduction interventions such as fentanyl test strip and naloxone distribution, as well as MOUD may improve safety. However, with a fentanyl-contaminated drug supply increasing risk for PWUO, safer opioid distribution of pharmaceutical-grade products and OPPs are needed to effectively address the burden of withdrawal, overdose, and fatality prevention within peer groups.

## Supporting information

S1 Interview Guide Qualitative interview guide assessing motivations for cannabis and opioid use. Attached is the semi-structured interview guide which includes domains, interview questions, and probes.
(DOCX)

S2 Codebook Descriptions of codes used for analysis. The attached qualitative codebook consists of 26 thematic codes groups by categories, sub-categories, and relevant quotes/memos in each code.
(DOCX)

S3 SRQR (Standards for reporting qualitative research). The qualitative reporting checklist is a set of 21 items that provide transparency and clear standards to understand qualitative data collection and analysis efforts. (DOCX)
