# Supplementary material for: “You take care of people, people will take care of you”: Moral Economies and an Unpredictable Drug Market
Source: PLoS One. 2025 Apr 2;20(4):e0320423. doi: 10.1371/journal.pone.0320423 (PMC11964208; doi:10.1371/journal.pone.0320423)
Supplement: S1 Interview Guide — Attached is the semi-structured interview guide which includes domains, interview questions, and probes. (DOCX) [file pone.0320423.s001.docx]

**PURPOSE**:

- Understand experiences with cannabis + opioids.
- Role cannabis + opioids play.
- Opportunities for cannabis + opioids use compared others substances

Background

- Tell me about the **first time** you used cannabis.
  - How old were you when you first used cannabis?
  - What were the circumstances + **context**?
- What was the **role** of cannabis when you were growing up?
  - How have your **motivations changed** over time?

Current

- **Cannabis**: Tell me about your **current use** of cannabis.
  - In what **contexts** do you use it?
  - **Where** and with **whom**?
  - What **role** does it play in your daily life?
  - What is your **primary delivery system** of cannabis?
  - Which route of **administration** do you most often use, and why?
    - **Probe**: pain mgmt, relaxation, pleasure, mental health, coping
- **Opioids:** Tell me about your **current use** of opioids.
  - In what **contexts** do you use it?
  - **Where** and with **whom**?
  - What **role** does it play in your daily life?
    - **Probe**: pain mgmt, relaxation, pleasure, mental health, coping

Pain Relief

- What are your views on the **therapeutic use** of cannabis + opioids?
  - Effective?
- What **conditions do you use** cannabis + opioids **to treat**?
- Do you use cannabis + opioids to **manage other health issues / symptoms**?

Emotional Regulation

- Have you ever used cannabis + opioids to **break out of / change the mood**?
- What conditions do you use cannabis + opioids **to treat / feel better**?
  - **Probe**: depression, anxiety, stress

Substitution

- Do you **prefer** one over the other—cannabis + opioids vs. other substances?
- How do you **compare** cannabis + opioids vs. other substances?
- Do you **feel a difference** when you use cannabis + opioids vs. other substances?
- Do you use cannabis + opioids in **combination** with other substances?
  - **Probe**: motivations, prior experiences, current use, stigmas, price, availability

Drug Effects

- Walk me through the last time that you used any kind of substance. What kind of substance(s) did you take?
  - What was the **context** when this happened?
  - Where did you get high?
  - Who did you get high with? Do you have a preference—alone or with others?
  - What kind of activities do you like to engage in while high?
  - What made you **decide to buy + use** cannabis on that occasion?
  - What did you **like** about the experience?
