# Supplementary material for: “You take care of people, people will take care of you”: Moral Economies and an Unpredictable Drug Market
Source: PLoS One. 2025 Apr 2;20(4):e0320423. doi: 10.1371/journal.pone.0320423 (PMC11964208; doi:10.1371/journal.pone.0320423)
Supplement: S2 Codebook — The attached qualitative codebook consists of 26 thematic codes groups by categories, sub-categories, and relevant quotes/memos in each code. (DOCX) [file pone.0320423.s002.docx]

**Opioid x Cannabis Study Codebook (26 codes)**

Research question

1. Cultural views of cannabis use, identifying beliefs, motivations, prior experiences, current use patterns in relationship to opioid use
2. Explore if cultural views of cannabis use align with changes in cannabis and opioid use patterns over time (substitution, escalation, diminishment, no change)

**Global codes (3)**

Opioid, Cannabis: only use when not clear in paragraph, when more context is needed for snippet

Good quote

**Motivations for cannabis / opioid use (6)**

**Using to alleviate mental health issues:** experiencing emotional benefits from cannabis / opioids beyond high

*Examples: using cannabis to alleviate anxiety [18]; [5]; mental health benefits 20, 21*

**Using to alleviate physical health issues:** experiencing physical benefit from cannabis / opioids beyond high

*Examples: pain relief; injury and using substance to help with pain*

**Using to mitigate withdrawal symptoms:** using cannabis to alleviate opioid withdrawal

*Examples: using cannabis to mitigate opioid withdrawal symptoms [5]; avoid comedown effects of opioids [21]*

**Using to help with sleep:** using cannabis to sleep

**Using to help with relaxing:** using cannabis to feel calm, relax

**Using to help with appetite:** using cannabis to support having appetite, digestion, stomach issues

**Changing patterns of opioid / cannabis use (6)**

**Changing use due to life event:** changing opioid use due to major life event or loss

*Examples: using after a major life change (death in the family, sexual abuse, etc.); injury at a young age and developed dependence from Rx opioids*

**Setting up a system for use:** describing structured cannabis / opioid use and processes of prioritization; organizing day on finding and timing their next dose

*Examples: trial and error of organizing time around varying needs [5]; organizing time around competing needs - family, work, substance use [5]; combining substances in daily routine [19]; starting day with opioid, get out of bed so not get sick; using opioids to give them energy/make them functional throughout the day*

**Planning finances around use:** describing needing to plan out finances in order to maintain use / avoid withdrawal

*Examples: stealing from drug store to sell items to get money to buy substances, hustles*

**Controlling opioid use with cannabis:** using less opioids or going longer without using opioids because of cannabis; offsetting opioid use with cannabis

*Examples: go longer without heroin; “fentanyl, IV it in the morning and if I weren't using cannabis I'd be smoking it within 2 to 3 hours; kind of forget about the fentanyl and keep riding along or going and doing my errands or doing what I need to do to get anything done.”/” And if I ever see anybody sick I tell them here, hit a joint. It’ll help you. And it does, it helps a lot. “*

**Changing use due to access:** decreasing/increasing cannabis or opioid use based on availability, access, ease of getting git

*Examples: using cannabis when opportunity presents itself, whichever products are available [19]; using substances based on availability [22]; not able to use in jail—forced to not be able to use; if I had a choice, I’d be using this kind of canna/opioid*

**Wanting to stop use:** describing why they would want to stop using opioids / cannabis

*Examples: wanting to wean off opioids; using methadone or suboxone*

**Identifying pattern and admin preferences (4)**

**Describing best forms of use**: knowing which opioids / cannabis (dosage, admin, kind, etc.) work best for them and how; personal patterns of use; why I use what I use

*Examples: using heroin to not get hooked on fentanyl, using fentanyl because tolerance is too high for heroin, acknowledging harmful effects of opioids; knowing which strains work best for them and how/not knowing which strains; not knowing which strain works best for them; using a syringe for heroin or fentanyl vs. smoking heroin or fentanyl*

**Comparing canna/opioid vs. other substance**: weighing pros/cons of opioid / cannabis use

Examples: *“but alcohol kills, it's way more addictive and you could die from alcohol withdrawals”;* differentiating risks of opioids rx vs street drugs [22]; comparing health risks of cannabis vs cigarettes [20]

**Fearing fentanyl:** expressing fears of overdosing; avoiding or not wanting to use fentanyl

*Example: using fentanyl w/out awareness [20]; avoiding but finding in other substances [23]*

**Having overdose due to fentanyl:** overdosing b/c unknown fentanyl in opioids

*Examples: “I wasn't a fentanyl user at all. I died on accident because somebody gave me something with fentanyl in it and it killed me; I thought it was heroin and they gave it to me as if it was heroin. I died six times*

**Sharing values and outlooks of opioid / cannabis use (4)**

**Exposure by significant person to substance use:** describing growing up and being exposed to problematic substance use through home/family relationships and reflecting on impact; connecting current adverse outcomes to family instability (previously “Normalization code)

*Examples: smoking with family; “we get along better when we smoke weed” observing family members/other adults using drugs; explaining how normal it was to use certain drugs in their household; exposure to heroin later in life by gf*

**Mistrusting pharma:** describing not wanting to use prescription meds and preferring cannabis or opioids

*Examples: “I’d rather do heroin it doesn’t cause those side effects like medication does”;* “And then there's also Kava, which is like the natural thing that does help.”

**Using canna/opioid to avoid healthcare**: describing why using cannabis/opioids to avoid healthcare; feeling care receiving is insufficient (cause of stigma from healthcare providers; previously “Using as healthcare”)

*Examples: opioids keep them “healthy”; don’t need to visit doctor as much/haven’t seen doctor in years*

**Exerting control over substance use:** describing taking on responsibility and determining boundaries of substance use

*Examples: taking care of self first so make sure have enough opioids before getting for friends;*

**Experiencing stigma and consequences from opioid / cannabis use (3)**

**Experiencing stigma from healthcare for use:** feeling stigma around substance use when engaging in health system / providers (effect)

*Examples: feeling discriminated by providers; receiving inaccurate care from providers b/c substance use; providers lacking awareness of physiological effects of substance use over time [23]; feeling judgment, misunderstanding about users from providers, losing access to methadone, experiencing precarity/ variable access [20] differing views of drug users in ER based on user vs hcp status [24]; “used to be you had to fight with a pharmacist to buy a bag of needles and they didn’t understand that it was not helping to increase the problem of drug addicts, it’s helping to save our lives”*

**Experiencing stigma / tension for using from others:** feeling stigma around opioid / cannabis use from family, peers, partner

*Examples: using cannabis around family, hiding use from others [21]; hiding opioid use from family, cannabis ok [18]; experiencing familial conflict due to withdrawal [5]; feeling judged by family for opioid use [19]; experiencing family/ financial tension with social substance use [20]; feeling misunderstood by those without experience using opioids [22]; Have to stay away from family when using heroin*

**Facing legal consequences for use:** facing / fearing legal conseq for use

using methadone to avoid further legal trouble [18]; facing legal consequences for cannabis paraphernalia [20]; fearing legal consequences of opioids [19, 18]
