# Supplementary material for: “You take care of people, people will take care of you”: Moral Economies and an Unpredictable Drug Market
Source: PLoS One. 2025 Apr 2;20(4):e0320423. doi: 10.1371/journal.pone.0320423 (PMC11964208; doi:10.1371/journal.pone.0320423)
Supplement: S3 SRQR — (DOCX) [file pone.0320423.s003.docx]

| No | Topic | Item |
| --- | --- | --- |
| **Title and abstract** | | |
|  | Title | “You take care of people, people will take care of you”: Moral economies and an unpredictable drug market |
|  | Abstract | Introduction  Fentanyl is the leading cause of opioid-related overdose deaths in the United States. Given the exogenous market shock of fentanyl and subsequent transition in the illicit opioid supply, our analysis sought to explore the social and relational experiences of people who use opioids (PWUO).  Methods  We conducted qualitative interviews with 30 PWUO (n=30) in Los Angeles, CA from July 2021 to April 2022. To be eligible for this study, participants had to report being 18 years of age or older and any self-reported opioid, cannabis, and injection drug use within the past 30 days. We used constructivist grounded theory to analyze the contexts that contribute to lived experiences surrounding opioid use behaviors within social networks.  Results  Within an unpredictable drug market contaminated by fentanyl, participants reported: 1) avoiding opioid withdrawal symptoms by sharing financial and material resources within social networks, 2) securing and cultivating known, predictable social ties to prioritize safe/ safer supply of opioids, and 3) avoiding and mitigating risk of overdose fatality by using opioids within peer groups.  Conclusions  Our findings emphasize that while peer support plays a critical role in safety within moral economies of PWUO, structural changes are needed to address the additional harms from an unregulated drug supply. Harm reduction interventions such as fentanyl test strip and naloxone distribution, as well as medication for opioid use disorders may improve safety. However, with a fentanyl-contaminated drug supply increasing risk for PWUO, safer opioid distribution of pharmaceutical-grade opioids and overdose prevention programs are needed to effectively address the burden of withdrawal, overdose, and fatality prevention within peer groups. |
| **Introduction** | | |
|  | Problem formulation | Within moral economies of unhoused PWUO, shared vulnerabilities emerging from shifting drug markets and mutual interdependencies of communities shape relationships and facilitate safer substance use. |
|  | Purpose or research question | This paper aimed to understand mutualistic strategies employed by PWUO to mitigate fentanyl-related health risks and reshaped amidst an increasingly unpredictable drug market. |
| **Methods** | | |
|  | Qualitative approach and research paradigm | Novel use of qualitative study design was used to study the contexts that contribute to lived experiences surrounding moral economies among PWUO.    Constructivist grounded theory methods were used for data collection and analysis throughout the study. |
|  | Researcher characteristics and reflexivity | E.E.G. has an MPH  S.S.G. has an AB and BS  A.J.D. has a PhD  J.H. has a PhD  R.N.B. has a PhD  R.C.C. has a PhD    A.J.D. is affiliated with the University of California San Francisco. All other authors are affiliated with the University of Southern California.    R.N.B., R.C.C., and J.H. are faculty members. E.E.G. is a full-time staff member. S.S.G. is also a PhD student and R.C.C. is one of her committee members. A.J.D. is a post doctorate fellow.    Doctoral students and staff researchers were trained in qualitative methods as part of a doctoral-level public health course and in fulfillment of learning qualitative methods to conduct their research projects or as part of lab meetings.    There was no prior relationship with participants. |
|  | Context | Interviews were conducted at two community sites, one affiliated with a syringe service program and another close to a methadone clinic in Los Angeles, California from July 2021 to April 2022. |
|  | Sampling strategy | We used convenience sampling to recruit and interview participants. |
|  | Ethical issues pertaining to human subjects | Research was approved by USC IRB (Study ID: HS-18-00624)  Written informed consent was provided before interviews. |
|  | Data collection methods | Qualitative, semi-structured, one-on-one interviews were conducted from July 2021 to April 2022. |
|  | Data collections instruments and technology | Interviews were conducted in-person using a voice recorder for later transcription. |
|  | Units of study | We recruited and interviewed 30 opioid using people who inject drugs. |
|  | Data processing | Transcriptions of interviews were done via an external transcriber who manually anonymized by removing identifying information about participants including names, cities, etc. Data integrity was verified by comparing audio from the interview to the transcription. Inconsistencies were edited accordingly. |
|  | Data analysis | ATLAS.ti was used to code themes and conduct analysis. |
|  | Techniques to enhance trustworthiness | We engaged in discussions of how codes were being interpreted by researchers to generate memos and compare observations, deviations, and track emerging ideas that would facilitate the development of theoretical concepts. These weekly analytic meetings spanned 2-3 hours per week for over 15 weeks and allowed us to engage in reflexive discussions, triangulate ideas between researchers, and construct the theories we were reporting. |
| **Results/findings** | | |
|  | Synthesis and interpretation | Participants described social support systems involving direct action to keep each other safe, which included: 1) sharing finances to head off withdrawal effects and mitigate financial changes which hinder opioid access, 2) sourcing from known and trusted people to avoid disruptions to and contamination of supply with fentanyl, and 3) preventing overdose by using with peers, monitoring, and administering overdose reversal agents. |
|  | Links to empirical data | This work uses participant quotes to link findings to the data. The following quotes are findings from various participants describing the role of moral economies among PWUO:     - *“[N]ot having fentanyl is absolutely the worst feeling in the world...if I don't have enough money... my friend will lend me some. He won't let me be sick... You take care of people, people will take care of you.” (Leo)* - *“I’ve stayed steady with the same dealer the whole time I’ve been doing fentanyl. So for like four years, I’ve gotten it from the same connect through this whole time. [It is advantageous because] if I need a front, I can get a front. Like my bags are fatter. Plus, he’s my friend. There's two of them. They both ended up being really cool people. So the main benefit is I made a couple of really good friends.” (Theo)* - *“[I use opioids] with my friends... I don't like to get high alone because I'm afraid if I do too much or something then I'm not going to be able to make it out if nobody's there with me so... I got the Narcan at the house and everybody knows where it's at, we know how to use it... because you never know with the Fentanyl.” (Ruby)* |
| **Discussion** | | |
|  | Integration with prior work, implications, transferability, and contributions to the field | This research yields important information about navigating the fentanyl-flooded opioid market by relying on social ties within moral economies. It also provides important insights in guiding interventions for alternative safety resources among opioid-using PWID. |
|  | Limitations | Data was collected at a methadone clinic and a syringe exchange site, it might overrepresent those engaged in treatment and harm reduction practices. The sample size and convenience approach to recruitment means results are not generalizable to other PWUO in Los Angeles or elsewhere. |
| **Other** | | |
|  | Conflicts of interest | The authors do not have any conflicts of interest to report. |
|  | Funding | The study was funded by NIDA R01DA046049-01A1S1. Ricky N. Bluthenthal and Siddhi S. Ganesh were supported by NIDA R01-DA046049. Siddhi S. Ganesh is also supported by Institute for Addiction Science pilot award PG1033682. The National Institute on Drug Abuse had no role in the design and conduct of the study; collection, management, analysis, and interpretation of the data, preparation, review, or approval of the manuscript, nor decision to submit the manuscript for publication. |
